# Optical Coherence Tomography Angiography to Distinguish Changes of Choroidal Neovascularization after Anti-VEGF Therapy: Monthly Loading Dose versus Pro Re Nata Regimen

**DOI:** 10.1155/2018/3751702

**Published:** 2018-02-04

**Authors:** Alexandra Miere, Hassiba Oubraham, Francesca Amoroso, Pauline Butori, Polina Astroz, Oudy Semoun, Elsa Bruyere, Alexandre Pedinielli, Manar Addou-Regnard, Camille Jung, Salomon Y. Cohen, Eric H. Souied

**Affiliations:** ^1^Department of Ophthalmology, University Paris Est Créteil, Centre Hospitalier Intercommunal de Créteil, Créteil, France; ^2^Clinical Research Center and Biological Resources Center, GRC Macula, Centre Hospitalier Intercommunal de Créteil, Créteil, France

## Abstract

**Purpose:**

To compare the qualitative and quantitative choroidal neovascularization (CNV) changes after antivascular endothelial growth factor (anti-VEGF) therapy in treatment-naïve and treated eyes with age-related macular degeneration (AMD) using optical coherence tomography angiography (OCTA).

**Methods:**

Consecutive patients with neovascular AMD underwent multimodal imaging, including OCTA (AngioPlex, CIRRUS HD-OCT model 5000; Carl Zeiss Meditec, Inc., Dublin, OH) at baseline and at three monthly follow-up visits. Treatment-naive AMD patients undergoing anti-VEGF loading phase were included in group A, while treated patients were included in group B. Qualitative and quantitative OCTA analyses were performed on outer retina to choriocapillaris (ORCC) slab. CNV size was measured using a free image analysis software (ImageJ, open-source imaging processing software, 2.0.0).

**Results:**

Twenty-five eyes of 25 patients were enrolled in our study (mean age 78.32 ± 6.8 years): 13 treatment-naïve eyes in group A and 12 treated eyes in group B. While qualitative analysis revealed no significant differences from baseline to follow-up in the two groups, quantitative analysis showed in group A a significant decrease in lesion area (*P* = 0.023); in group B, no significant change in the lesion area was observed during anti-VEGF therapy (*P* = 0.93).

**Conclusion:**

Treatment-naïve and treated eyes with CNV secondary to neovascular AMD respond differently to anti-VEGF therapy. This should be taken into account when using OCTA for CNV follow-up or planning therapeutic strategies.

## 1. Introduction

Paradigms concerning age-related macular degeneration have been shifting rapidly over the last decade, due to both therapeutic advances (i.e., antivascular endothelial growth factor therapy for neovascular AMD) and retinal imaging advances; among which, spectral domain optical coherence tomography, enhanced depth imaging optical coherence tomography, and optical coherence tomography angiography are noteworthy. OCTA is a new retinal imaging technique providing information on the actual location of the choroidal neovascularization, on various abnormalities of the retinal and choroidal microvasculature in a noninvasive manner [1–3]. Its exponential growth over the last few years asserts for its usefulness not only in an academic setting (giving insights into the pathogenesis of several macular disease), but also in a clinical setting [4–6].

Nevertheless, SD-OCT is a key element in detecting recurrences, and treatment decisions are frequently based solely on this noninvasive imaging method [7–9]. Classically, recurrences of CNV are defined on SD-OCT by a myriad of signs, ranging from subretinal or intraretinal fluid, subretinal hyperreflective material, or changes in the pigment epithelial detachment's height [7–11]. When comparing these signs to fluorescein angiography (where the presence of late leakage or dye pooling is the central element in defining the presence of CNV) [12, 13] or indocyanine green angiography (which allows a clear visualization in its late frames of occult CNV) [14], the consequent conclusion is that of a growing complexity and greater refinement of the diagnosis provided by retinal imagery over time. However, while the signs provided by SD-OCT or conventional angiography are used to define activity of CNV to this day in a clinical setting, the concept of CNV activity itself involves more complicated, intricate mechanisms than previously thought [15]. Moreover, recent fundamental research studies have concluded that, in addition to elevated VEGF levels, dysregulated inflammation and autoimmunity play an essential role in CNV pathogenesis [16, 17].

In parallel to the complexity of CNV seen as either/or a disease or a compensatory response [15, 18], the management of neovascular AMD has been revolutionized by a series of pharmaceutical agents, such as ranibizumab (Lucentis; Genentech Inc., South San Francisco, CA), [19] aflibercept (Eylea; Regeneron Inc., Tarrytown, NJ), [20], and the off-label option bevacizumab (Avastin; Genentech Inc.), [21] which all inhibit VEGF-A. Moreover, multiple management strategies have been employed for anti-VEGF therapy, such as monthly dosing, every other month dosing after 3 initial monthly loading phase [20], and monthly loading phase followed by pro re nata (PRN) retreatment on evidence of exudative disease activity [22], as well as treat and extend regimens [23].

In our study, we aimed to compare the OCTA characteristics (using both qualitative and quantitative criteria) of eyes with neovascular AMD undergoing the monthly loading phase (treatment-naïve at baseline) versus eyes undergoing an as needed (PRN) regimen (previously treated at baseline).

## 2. Methods

### 2.1. Study Population

All consecutive patients of at least 60 years, presenting at the Montargis Eye Clinic (France) and Department of Ophthalmology of University Paris Est, in Creteil, France, between August 2016 and January 2017, diagnosed with treatment-naive or treated neovascular AMD were enrolled in this prospective comparison of qualitative and quantitative features of CNV undergoing anti-VEGF therapy in the loading phase (treatment-naive patients) and pro re nata regimen (treated patients). Visual outcomes from baseline to final visit were also assessed.

Diagnosis of type 1 and type 2 CNV was based on fundus biomicroscopy, fluorescein angiography (FA), and SD-OCT (CIRRUS HD-OCT model 5000, Carl Zeiss Meditec Inc., Dublin, California, USA). Only eyes with baseline presence of subretinal hyperreflective material (SHRM), intraretinal or subretinal fluid (SRF), and pigment epithelial detachment (PED) were included.

Two distinct groups were emerged: treatment-naive AMD patients undergoing anti-VEGF loading phase were included in group A, while previously treated patients undergoing PRN were included in group B.

Exclusion criteria consisted in type 3 neovascularization, media opacities, evidence of diabetic retinopathy or any other macular or retinal vascular disease, signs or history of central serous chorioretinopathy, and hereditary retinal dystrophy. Patients with poor quality images in OCTA were also excluded from the analysis. The study was performed in agreement with the Declaration of Helsinki for research involving human subjects and the French legislation. Our local Institutional Review Board approval was obtained for this study.

### 2.2. Study Protocol

At baseline, each enrolled patient underwent a complete ophthalmic examination including best-corrected visual acuity (BCVA), slit-lamp examination, fundus biomicroscopy FA, SD-OCT, and OCTA. OCTA was performed through AngioPlex CIRRUS HD-OCT model 5000 (Carl Zeiss Meditec Inc., Dublin, California, USA). OCTA was performed in all patients using a scanning area of 3 × 3 mm, centered on the fovea.

AngioPlex uses optical microangiography, which is an imaging technique that produces tridimensional images of dynamic blood perfusion at an imaging depth up to 2.0 mm [24, 25]. The instrument has an A-scan rate of 68,000 scans per second, using a superluminescent diode centered on 840 nm. The resultant 3 × 3 angio cube contains 245 B-scan slices repeated up to 4 times at each B-scan position. Each B-scan is made up of 245 A-scans; each A-scan is 1024-pixel deep [26].

Each included patient underwent 3 monthly visits following inclusion. BCVA, SD-OCT, and OCTA were performed during these follow-up visits. For patients included in group A (treatment-naive), an intravitreal injection was performed after the follow-up visit, while for patients included in group B, treatment indication was based on the presence/absence of classical activity signs on SD-OCT.

Two independent masked readers performed a qualitative and quantitative assessments. Qualitative analysis of OCTA ORCC segmentation images at baseline and at each follow-up visit consisted in morphological criteria from recent literature, [3–6, 27–29] such as presence/absence of a high-flow network, presence/absence of tiny ramifications, presence/absence of feeder vessel, presence/absence of an anastomotic arcade, presence/absence of a dark halo, presence/absence of flow void, and presence/absence of arteriolized vessels. All images were analyzed by the two independent readers using the outer retina to choriocapillaris (ORCC) slab, which allows a good visualization of type 1 and type 2 CNV important features [30]. Quantitative analysis of CNV size was performed on the same slab, using free image analysis software (ImageJ, open-source imaging processing software, 2.0.0-rc-43/1,51 K).

Quantitative and qualitative changes on OCTA were then compared between the two groups and correlated with best-corrected visual acuity (BCVA) and exudation signs on structural spectral domain (SD-OCT).

### 2.3. Statistical Analysis

Statistical data analysis was carried out using the STATA software (version 13.0, STATACORP LP, College Station, TX, USA). Qualitative variables were described in percentages, and quantitative variables were described by mean with standard deviation or by median with interquartile range. Intragroup serial comparisons of categorical variables at baseline and month 3 were carried out using the Wilcoxon signed-rank test. Intragroup serial comparisons of continuous variables at baseline and follow-up were carried out using the Exact McNemar signification probability test. Intergroup serial comparisons were carried out using Mann–Whitney test for categorical variables and Pearson's chi-square for continuous variables. Cohen's kappa coefficient was used to measure the interuser agreement for qualitative items on OCTA at baseline and follow-up. The chosen level of statistical significance was *P* < 0.05.

## 3. Results

### 3.1. Demographic Information

Twenty-five eyes of twenty-five patients were enrolled in our study (13 females, 12 males, mean age 78.32 ± 6.8 years). The final cohort for analysis consisted of 2 groups:

- Group A: 13 treatment-naïve eyes of 13 patients (7 females, 6 males, mean age 80.3 ± 7.57 years) that underwent monthly loading phase.
- Group B: 12 previously treated eyes (6 females, 6 males, mean age 76.16 ± 5.37) by a mean of 7.16 ± 2.94 anti-VEGF intravitreal injections. Mean time from the diagnosis was 13.1 ± 2.56 months.

BCVA for the cohort as a whole at baseline was 60.64 ± 16.88 letters (Snellen equivalent 20/64). Baseline BCVA for eyes included in group A was 57.54 ± 20.52 letters (Snellen equivalent 20/80), while in group B baseline BCVA averaged 64 ± 11.78 letters (Snellen equivalent 20/50). Mean follow-up for both groups was 3 months.

### 3.2. Qualitative Assessment: Morphological OCTA Changes in the Monthly Loading Phase (Group A) versus PRN Phase (Group B) Eyes

At each visit, a high flow network in the ORCC slab was visualized in all cases (25/25). There was no statistical significant change in either of the above-mentioned features (presence/absence of a high-flow network, ramifications, feeder vessel, anastomotic arcade, dark halo, feeder vessel, and arteriolized vessels) in groups A and B, respectively, from baseline to month 3 (*P* ranging from 0.08 to 1).  Table 1 shows the detailed morphological assessment of OCTA ORCC segmentation images.

### 3.3. Quantitative Assessment: OCTA Lesion Area Changes in the Monthly Loading Phase (Group A) versus PRN Phase (Group B) Eyes

**Figure 1:**
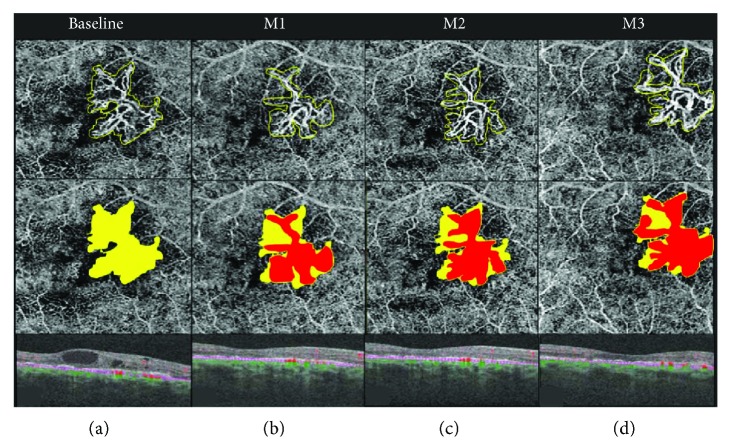
OCTA follow-up of treatment-naïve choroidal neovascularization (CNV) associated to neovascular age-related macular degeneration (AMD) during the monthly loading anti-VEGF phase. Each column represents a visit; the upper row represents the quantification of CNV size on the outer retina to choriocapillaris (ORCC) slab; the lower row represents the comparison between baseline lesion area (yellow) and follow-up CNV area (red). Quantitative analysis demonstrated that at baseline, the area in the ORCC segmentation averaged 1.06 mm^2^. Under anti-VEGF therapy, this area decreased significantly, to 0.55 mm^2^, at month 1 (M1). At month 2 (M2), the area averaged 0.72 mm^2^, while at month 3 (M3), at the end of the loading phase, the CNV area was 0.87 mm^2^. Indeed, the CNV area decreased from baseline to month 3 by 17.39%. This decrease has been noticed on the overall cohort of treatment-naïve in a statistically significant manner (*P* = 0.02, Figure 3).

**Figure 2:**
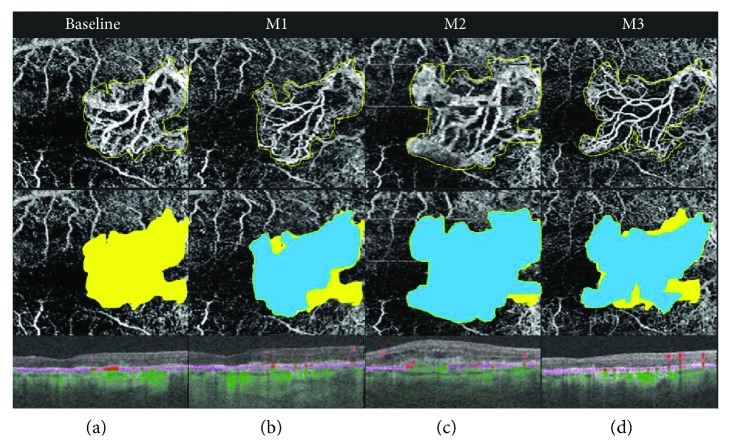
OCTA follow-up of previously treated choroidal neovascularization (CNV) associated to neovascular age-related macular degeneration (AMD) during pro re nata (as needed) regimen. Each column represents a visit; the upper row represents the quantification of CNV size on the outer retina to choriocapillaris (ORCC) slab; the lower row represents the comparison between the baseline lesion area (yellow) and follow-up CNV area (blue). Quantitative analysis demonstrated that at baseline, the area in the ORCC segmentation averaged 1.93 mm^2^. Under anti-VEGF therapy, this area decreased to 1.85 mm^2^, at month 1(M1). At month 2 (M2), the area increased to 2.92 mm^2^, while at month 3 (M3), at the last follow-up visit, the CNV averaged 2.45 mm^2^. Indeed, the CNV area slightly increased from baseline to month 3 by 26.94%. This expansion of the CNV size in treated patients has been noticed on the overall cohort (*P* = 0.94, Figure 3).

**Figure 3:**
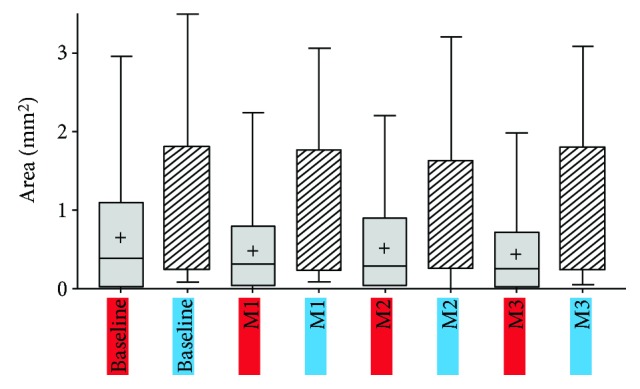
Box-and-whisker plot showing the comparison of CNV size between groups A and B. Group A (grey box on the plot; group A consists of treatment-naïve patients undergoing monthly loading phase) and group B (stripped box; group B consists of treated patients undergoing treatment according to a pro re nata regimen). Note that at baseline the treatment-naïve patients (grey box) had a smaller lesion size than the patients that had already been treated by anti-VEGF (stripped box). During the loading phase, treatment-naïve patients showed a statistically significant decrease in CNV size (*P* = 0.02), while the treated patients undergoing PRN regimen demonstrated a slight increase in the lesion size (*P* = 0.93) at month 3.

In the group undergoing monthly loading dose (group A), there was a statistically significant decrease in the lesion area, from 0.66 ± 0.84 mm^2^ at baseline to 0.23 ± 0.3 mm^2^ at month 3 (*P* = 0.02, Wilcoxon signed-rank test). However, in the previously treated group now undergoing PRN regimen (group B), the lesion area slightly increased, from 0.94 ± 1.06 mm^2^ at baseline to 0.98 ± 1 mm^2^ at month 3 (*P* = 0.93, Wilcoxon signed-rank test).  Table 2, as well as Figures 1, 2, and 3, shows these changes.

### 3.4. Best-Corrected Visual Acuity Changes in the Monthly Loading Phase (Group A) versus PRN Phase (Group B) Eyes

At baseline, there was no statistically significant difference in terms of BCVA between the two groups (*P* = 0.44, Mann–Whitney test). However, during follow-up, there was a statistically significant improvement in group A from 57.54 ± 20.52 letters at baseline to 65.33 ± 22.33 letters at month 3 (*P* = 0.009, Wilcoxon signed-rank test). Conversely, in group B, there was no statistically significant change in BCVA, which averaged 64 ± 11.78 letters at baseline and 61.25 ± 26.36 letters at month 3 (*P* = 0.4, Wilcoxon signed-rank test).

### 3.5. Reproducibility for Qualitative and Quantitative Analyses

For the criteria used in the qualitative analysis of OCTA images, interuser agreement was 94.9% (Cohen's K coefficient 0.91, standard error 0.14). For the quantitative criteria, interclass correlation coefficient (ICC) was high for the lesion area measurement, averaging 0.97 (CI 95% 0.96–0.98).

## 4. Discussion

In our study, we showed the difference in terms of qualitative and quantitative features on OCTA between a group of treatment-naïve patients undergoing monthly loading phase and a group of previously treated patients undergoing PRN regimen. While for the qualitative chosen criteria there was no statistically significant difference between the two groups, we note a higher prevalence of features associated with vascular immaturity (the presence of capillary sprouting, harboring the aspect of tiny ramifications within the neovascular membrane) [6, 29, 31] at baseline in the treatment-naïve group (group A) when compared to the treated group (group B). Conversely, in group B, the presence of arteriolized vessels, suggesting vascular maturity, [29] was higher (5/12) at baseline and progressively increased during follow-up.

Concerning lesion size as measured on the ORCC segmentation on OCTA images in the two groups, there was a significant decrease of the neovascular membrane size from baseline to month 3 in group A, suggesting that lesion size can be considered as a marker of therapeutic response to anti-VEGF in treatment-naïve nAMD patients. However, in group B, which had been diagnosed with nAMD 13.1 ± 2.56 months earlier and thus treated by a mean of 7.16 ± 2.94 anti-VEGF intravitreal injections, the neovascular membrane's area slightly increased over time (from 0.94 ± 1.06 mm^2^ at baseline to 0.98 ± 1 mm^2^ at month 3), suggesting that, for treated patients with medium to longstanding diagnosis of nAMD, a lesion size might be a questionable marker in assessing therapeutic response. As for the best-corrected visual acuity, there was a significant improvement (*P* = 0.009) only in group A, while in group B, the visual acuity did not improve significantly (*P* = 0.40) over the 3 months of follow-up.

Follow-up of nAMD by means of OCTA has been emphasized in the last years in the recent literature. Lumbroso et al. described, in 2015, the morphological changes within the type 2 neovascular membrane in neovascular AMD undergoing anti-VEGF treatment, suggesting the presence of patterns of cyclic CNV variations during follow-up and stating that the CNV cycle is 62 days long. [31]. Furthermore, Coscas et al. estimated that different CNV patterns detected on OCTA had a correspondence with treatment decisions by multimodal imaging on their cohort [32]. Moreover, Muakkassa et al. approached this characterization of treatment-naïve CNV after anti-VEGF treatment on a larger spectrum of retinal pathology (from naMD to neovascularized idiopathic macular telangiectasia or multifocal choroiditis), also measuring the greatest linear dimension and area of these CNV. Their results showed that both of these categorical variables decreased (by 23.6% for greatest linear dimension and 29.8% for area, resp.) after anti-VEGF treatment [4]. Huang et al. revealed that the quantitative measurements of CNV flow area revealed a shutdown of flow 2 weeks after the injection, with reappearance of the CNV vascular channels in week four and actual exudative signs in week 6 [33].

On one hand, OCTA follow-up of CNV mentioned above strongly suggested the existence of a lifecycle of the CNV, with area being one of the markers for treatment response [6]. Spaide et al. made significant observations on the evolution of treated eyes with advanced nAMD using OCTA, by focusing on two vascular phenomena, arteriogenesis and angiogenesis, in an attempt to explain the longstanding persistence and change in morphology (vessel arteriolization) in patients with longstanding nAMD [29].

**Figure 4:**
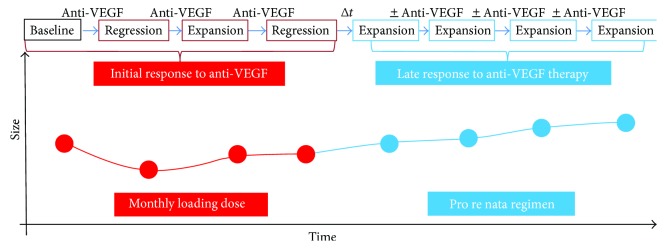
Illustrative drawing of suggested types of response to antiangiogenic treatment in CNV associated to neovascular AMD. Treatment-naïve, immature CNV, undergoing monthly loading phase, is depicted in red. Previously treated CNV, undergoing PRN, is depicted in blue. In the treatment-naïve eyes, there will be an initial response to treatment, characterized by regression (contraction) of the lesion area after each intravitreal injection (red dots). This initial response to anti-VEGF may be replaced after a variable amount of time(*Δt*) in an as needed protocol (PRN) by a late response in which, despite the disappearance of exudative signs of CNV, their area will continue to expand (blue dots). The latter type of response corresponds to a mature CNV.

Taking these observations into consideration altogether with the variation in lesion area in our two groups, there may actually be two different responses to anti-VEGF therapy depending on disease duration (and thus of CNV immaturity/maturity). We hypothesize that in immature, treatment-naïve CNV there will be an initial response to treatment, characterized by regression (contraction) of the lesion area; this initial response to anti-VEGF shall be replaced in time by a late response, in which despite the disappearance of exudative signs of CNV, their area will continue to expand. The latter type of response corresponds to a mature CNV. The two types of response are illustrated in Figure 4.

Our study has several limitations, among which the most important are the small sample size and the heterogeneity of our cohort. Last but not least, artifacts are still an important issue in OCTA technology. The slab we used to analyze OCTA images, given the hyperreflectivity of the retinal pigment epithelium (RPE), includes projection artifacts from the overlying superficial capillary plexus. Thus, we have analyzed images with and without projection artifact in order to correctly delineate the neovascular membrane.

In conclusion, our results suggest that there are two types of treatment responses to anti-VEGF, depending upon disease duration. Our data has two main corollaries: firstly, on the validity of a PRN treatment regimen, leaving therapeutic windows that could favor the growth and possibly the maturation of CNV. Secondly, on the way we should assess treatment response by OCTA in a clinical setting, taking into account disease duration and previous treatment. Of course, prospective studies on larger cohorts with a long follow-up should be performed in order to validate our findings.

## Figures and Tables

**Table 1:** Comparison of categorical variables between baseline and month 3 in groups A (treatment-naive patients undergoing monthly loading phase) and B (treated patients undergoing PRN regimen). Note that there is no statistically significant morphological change between baseline and month 3 (computed *P* value) in either group.

	Categorical variables	M0	M1	M2	M3	Significance^∗^
Group A *N* = 13	High-flow network	13	13	13	13	*P* = 1
	Ramifications	11	7	6	3	*P* = 0.25
	Feeder vessel	3	3	1	3	*P* = 1
	Anastomotic arcade	1	1	0	1	*P* = 0.5
	Dark halo	4	4	2	4	*P* = 1
	Flow void	1	1	1	1	*P* = 0.5
	Arteriolized vessels	1	1	2	3	*P* = 0.8

Group B *N* = 12	High-flow network	12	12	11	11	*P* = 1
	Ramifications	10	8	6	6	*P* = 0.13
	Feeder vessel	4	4	4	4	*P* = 1
	Anastomotic arcade	4	2	2	2	*P* = 1
	Dark halo	8	9	8	5	*P* = 0.25
	Flow void	2	1	0	3	*P* = 0.5
	Arteriolized vessels	3	4	4	6	*P* = 0.25

^∗^Exact McNemar significance probability; *P* value computed between baseline and month 3.

**Table 2:** Comparison of continuous variables between baseline and month 3 in groups A and B. Note that the CNV area decreased significantly in group A (*P* = 0.02) from baseline to month 3 and that BCVA improved in a statistically significant manner in group A from baseline to month 3 (*P* = 0.009).

	Continuous variables	Baseline	Month 1	Month 2	Month 3	Significance^∗^
Group A *N* = 13	CNV area (mm^2^)	0.66 ± 0.84	0.49 ± 0.65	0.28 ± 0.42	0.23 ± 0.3	*P* = 0.02
	BCVA (letters)	57.54 ± 20.52	57.14 ± 24.58	58.07 ± 26.05	64.15 ± 22.32	*P* = 0.009
Group B *N* = 12	CNV area (mm^2^)	0.94 ± 1.06	0.92 ± 0.99	0.96 ± 1.01	0.98 ± 1.00	*P* = 0.93
	BCVA (letters)	64 ± 11.78	64 ± 17.67	56 ± 25.25	62.66 ± 26.36	*P* = 0.4

^∗^Wilcoxon signed-rank test, *P* value computed between baseline and month 3.
